# Empowering Veterinary Herd Health Management: Insights into Education, Implementation, and Regulation Across Europe

**DOI:** 10.3390/vetsci11110528

**Published:** 2024-10-30

**Authors:** Marina Marić, Vidhi Manghnani, Jarkko K Niemi, Tarmo Niine, Nancy De Briyne, Wiebke Jansen

**Affiliations:** 1 Federation of Veterinarians of Europe, Rue Victor Oudart 7, 1030 Brussels, Belgium; mmaric2@vef.hr (M.M.); nancy@fve.org (N.D.B.); 2 Faculty of Veterinary Medicine, University of Zagreb, Ul. Vjekoslava Heinzela 55, 10000 Zagreb, Croatia; 3 University of Veterinary Medicine Budapest, István u. 2, 1078 Budapest, Hungary; 4 Bioeconomy and Environment Unit, Natural Resources Institute Finland (Luke), 60320 Seinäjoki, Finland; jarkko.niemi@luke.fi; 5 Institute of Veterinary Medicine and Animal Sciences, Estonian University of Life Sciences (EMU), Kreutzwaldi 62, 51006 Tartu, Estonia; tarmo.niine@emu.ee

**Keywords:** herd health management, preventive veterinary medicine, veterinary, integrated animal health management

## Abstract

In recent years, integrated veterinary herd health management (VHHM), improving animal health, welfare, food safety, public health, farm management, and economics, has become ever-more important in the European Union due to structural and regulatory advancements. A mixed-methods study analyzed the current state and gaps in VHHM education and training across Europe. Data from 41 European veterinary institutions showed that 83% (*n* = 34/41) offered VHHM education, either as standalone courses and/or integrated into other subjects. An online survey for veterinarians, primarily working with cattle, poultry, pigs, and small ruminants (75%; *n* = 86/114), assessed perceived training knowledge gaps and needs. The majority of respondents were unaware of VHHM training programs (69.4%, *n* = 43/62) or projects (59.6%, *n* = 59/99) and reported the biggest gaps in VHHM training certification (14%, *n* = 7/49), followed by soft skills (12%, *n* = 6/52) and demonstrating VHHM benefits to their clients (9%, *n* = 5/53). To conclude, while undergraduate VHHM education was generally adequate, enhancing post-graduate certified multi-species training opportunities, incl. soft skills VHHM training were perceived as essential. Our findings also underscored the urgent need for robust economic evaluations of preventive strategies across various animal species to allow veterinarians to better demonstrate VHHM’s direct and indirect benefits to farmers.

## Simple Summary

Integrated veterinary herd health management improves animal health and welfare, public health, farm management, and economics. This study explores current veterinary herd health management education and training opportunities for veterinary students and professionals in Europe, identifying gaps, needs, and areas for improvement. An analysis of 41 European veterinary education establishments found that 83% (*n* = 34/41) taught veterinary herd health management in their curriculum, either as a standalone course and/or integrated into other subjects. However, while undergraduate education was generally adequate, coverage of more species (e.g., aquaculture) and enhancing soft skills instruction were identified as beneficial. A survey of primarily veterinarians working with cattle, poultry, pigs, and small ruminants assessed training gaps and needs in veterinary herd health management, mainly for soft skills and training certification. They reported challenges in effectively communicating its benefits to farmers and a lack of soft skills needed to promote the concept effectively. The majority of participating veterinarians were not aware of existing training programs (69.4%) and ongoing projects (59.6%) on this topic. To conclude, while undergraduate education on veterinary herd health management is generally adequate, enhancing post-graduate certified multi-species training opportunities, incl. soft skills training, was perceived as essential.

## 1. Introduction

In recent decades, the importance of veterinary herd health management (VHHM) has grown significantly in veterinary medicine due to profound structural changes [1]. In Europe, the number of farms has decreased considerably, while herd sizes have increased [2]. Between 2001 and 2021, the total number of livestock in the European Union (EU)—including pigs, cattle, sheep, and goats—fell by approximately 11.5%, from 326 million to 289 million. This trend resulted in animals being kept in a spatially more concentrated manner, which heightens the risk of disease transmission and necessitates a greater emphasis on biosecurity and preventive medicine [3]. At the same time, societal pressures to improve animal welfare and protect the environment are increasing. Consequently, the focus has shifted gradually from curing the individual to herd-level care. It was shown that the percentage of veterinarians practicing with companion animals increased from 67% in 2018 to 71% in 2023, while the percentage of veterinarians dealing with livestock species in the same period declined: cattle (26% to 23%, resp.), small ruminants (21% to 18%, resp.), and pigs (14% to 13%, resp.) [4]. Specific data from the United Kingdom (UK) revealed that only 12% of veterinarians worked in mixed practice [5], down from 22% in 2010. In the United States (US), the American Veterinary Medical Association (AVMA) 2020 report on the economic state of the veterinary profession shows that in the USA in 2021, only 1.4% of veterinarians worked with food-producing animals only and 6.5% worked with mixed food and companion animals, versus 71.7% working with purely companion animals [6]. The US report also showed that only 2.7% of new graduates were attracted by food animal-exclusive and food animal-predominant practice. VHHM was conceptualized in the 1990s as a multisectoral approach that integrates animal health, food safety, animal welfare, and public health with farm management and economics based on advanced knowledge of animal husbandry, nutrition, disease epidemiology, and preventive veterinary medicine [7,8]. Defined by combining preventive veterinary activities with the analysis of relevant data, VHHM enables veterinarians to provide informed advice to animal owners, aiming to optimize the health, welfare, and productivity of their animals [9,10]. VHHM focuses inherently on a group of animals of the same type that live and feed together [11]. While ‘herd’ mostly refers to cattle and pigs, VHHM is an equally crucial concept for other groups of animals, such as poultry and small ruminant flocks, and even animal shelter populations. Building upon population medicine, VHHM emphasizes the health and welfare of animal groups rather than individual animals, with a strong focus on preventive measures and data-driven decision-making. VHHM plans must be tailored to the specific needs of the establishment to effectively address the unique challenges faced by each farm or facility.

**Figure 1:**
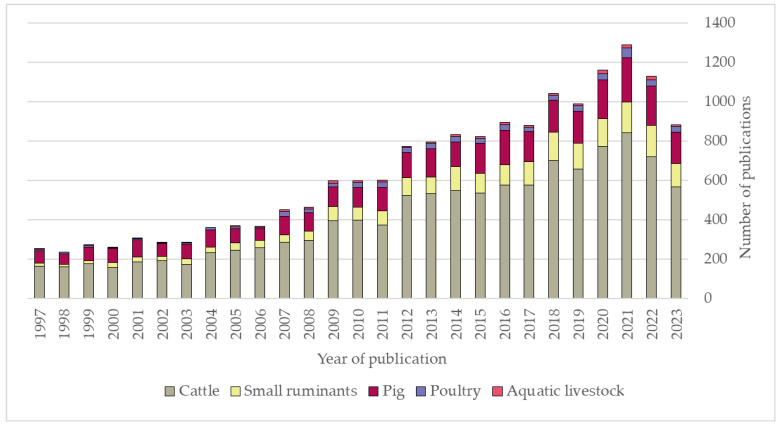
Number of publications per year from a Web of Science search for articles on herd health within specific animal groups, taken on 29 July 2024. Search terms used: herd health AND (1) (cattle OR dairy OR cow OR cows OR calf OR calves), (2) (small ruminant OR small ruminants OR goat OR goats OR sheep OR lamb OR lambs OR kid), (3) (pig OR pigs OR sow OR sows OR hog OR hogs OR swine), (4) (poultry OR chicken OR chickens OR duck OR ducks OR broiler OR broilers) and (5) (fish OR fishes or aquatic). In total, we found 17,211 publications, of which approximately 65% were on cattle.

To give readers a brief overview of the topic scope, a structured web search string conducted in the web interface of Web of Science (WoS) using search keys ((herd) AND various terms for cattle, small ruminants, pigs, poultry, and aquatic livestock) AND (herd health) showed an annually increasing number of articles. We limited the data to include only articles, review articles, and book chapters published during 1997–2023. In total, we found 17,211 publications, of which approximately 65% were on cattle (Figure 1).

These factors highlight the need for a multisectoral approach that links animal health, welfare, veterinary public health, and the development of farm-specific VHHM plans in a ‘prevention is better than cure’ approach. These changes cumulated in the adoption of mandatory preventive animal health visits as laid down in Article 25 of Regulation (EU) 2016/429 (Animal Health Law, AHL) and directly applicable in all EU Member States (MS) as of 2021. The AHL laid down requirements for all operators to ensure that all of their establishments keeping animals receive regular animal health visits from a veterinarian. However, extrinsic (e.g., available knowledgeable and sufficiently trained workforce and legislative framework) and intrinsic factors (e.g., motivation and attitude) hamper its implementation, which has been shown to vary greatly between sectors and countries [12].

The aim of our study was to identify approaches that are used to address the rising significance of VHHM in veterinary undergraduate and postgraduate education and veterinary practice.

## 2. Materials and Methods

### 2.1. Undergraduate VHHM Education

The European Veterinary Education Establishments’ (VEEs) integration of veterinary herd health management education into their undergraduate curricula was mapped qualitatively and quantitatively based on their results of the European System of Evaluation of Veterinary Training (ESEVT), the professional peer evaluation system for veterinary education establishments and their accreditation. The ESEVT is a Europe-wide, profession-specific evaluation system that ensures efficient preparation of veterinary students and an internationally recognized system for the evaluation of veterinary undergraduate training worldwide. A total of 41 evaluation reports of VEEs issued by the ESEVT between 2018 and 2022 were examined between January 2023 and March 2023 to assess VEEs’ compliance with ESEVT standard operating procedures (SOPs) of education and the level of VHHM education [13]. The assessment criteria were (i) accreditation status due to insufficient VHHM education, (ii) number and integration of VHHM subjects offered to students (practical/theoretical and species coverage), (iii) number of visited livestock units, and (iv) the peer evaluation remarks on the VHHM education.

### 2.2. Veterinary Practitioners’ Training

An online survey was set up to map and identify training opportunities and assess the gaps and needs in VHHM training in the EU MS and EFTA countries (Iceland, Liechtenstein, Norway, and Switzerland). The survey was distributed using a snowball sampling method to the 39 member organizations of the Federation of Veterinarians of Europe (FVE) to forward the survey to their individual members and the participants of COST action CA20103 BETTER (Biosecurity Enhanced Through Training Evaluation and Raising Awareness). One of these COST action working groups aims to identify training needs through the evaluation of existing training materials and to develop new courses, therefore increasing the number of trained professionals. The FVE joined forces with the COST action BETTER on the similar question of effective mapping of resources about current VHHM and biosecurity projects, courses, and virtual training that are available in local languages or with restricted access.

Prior to its distribution, the online survey was reviewed and approved on 10 July 2023 by the data protection officer at the Estonian University of Life Sciences and was open from 11 July to 12 September 2023. The survey did not collect any personal information. A stepwise approach was applied to the question sequence, ‘Do you know any ongoing or recent projects that are related to herd health management in animals?’. First, a list of ongoing projects was presented to indicate their knowledge of the listed projects, and secondly, respondents were able to name other projects that were not listed in the following question. Perceived training gaps were indicated on a four-point scale from ‘there is no gap’ to ‘very big gap’ (Supplementary Table S1).

To enable responses in multiple languages, the survey was translated into Albanian, German, Italian, Portuguese, Spanish, and Turkish, in addition to English. The survey was created using the online survey tool QuestionPro (QuestionPro. Inc., Austin, TX, USA). Responses related to VHHM training were validated and completed using the web search engine “Google” and predefined criteria, with supplementary information gathered from relevant web pages. Responses were extracted to Microsoft Excel^®^ v2410 for further descriptive analysis, summarizing the frequencies and dispersion of the data set.

### 2.3. Limitations

The data used with respect to undergraduate VHHM education, derived from the evaluation reports of the different VEEs, provide only a summary view of the situation as described by the visiting experts. This could have resulted in aspects of VHHM education within the VEEs missing from the reports, potentially leading to an underestimation of VHHM educational opportunities. The non-probability snowball sampling over the limited survey time of 2 months covering the summer period of the online survey on VHHM made it difficult to determine the representativeness of the sample, the sampling error, or to generalize inferences about the studied entities based solely on the questionnaire responses obtained. We acknowledge that the responses may reflect the views of those who are more proactive in promoting VHHM, potentially skewing the results due to selection bias. Furthermore, due to the limited number of responses in the survey received for the predetermined species, the results cannot be generalized and, therefore, should be interpreted with caution. The interviews on post-graduate training opportunities may have introduced subjectivity and selection bias and will have resulted in missing important national/regional training opportunities. Despite these limitations, we believe that our survey provides valuable insights into the key opportunities and challenges related to the education and application of VHHM in veterinary undergraduate education, postgraduate training, and practice.

## 3. Results

### 3.1. Undergraduate VHHM Education Opportunities

From the 41 VEE reports evaluated, *n* = 34 (83%) VEEs complied fully with the ESEVT SOP requirements of VHHM education, whereas *n* = 5/41 VEE (12%) received critical remarks on the quality or quantity of practical teaching and *n* = 2/41 VEE (5%) received critical remarks on the theoretical teaching of VHHM.

The majority (*n* = 30/41, 73%) covered the basic principles of VHHM integrated into other courses through relevant subjects in the mandatory core curriculum such as animal husbandry and breeding, animal nutrition, epidemiology, microbiology and infectious diseases, parasitology, internal medicine and nutritional, veterinary public health, preventive veterinary medicine, and animal disease legislation. The remaining 27% of VEEs (*n* = 11/41) had in addition specific VHHM lectures as a separate course. Few VEEs covered companion animal shelter management and aquatic animals (each *n* = 5/41, 12%) (Table 1).

### 3.2. Veterinary Practitioners’ Training Opportunities

The survey had 114 respondents, with the majority categorizing themselves as veterinarians (75%; *n* = 86/114), *n* = 23 as other stakeholders such as trainers or consumers (20.2%), *n* = 4 as farmers (3.5%), and 1 undergraduate student. Of those veterinarians who indicated their main field of practice (*n* = 65), the majority worked with cattle (47.7%; *n* = 31/65), followed by poultry (15.4%, *n* = 10/65), pigs (13.9%, *n* = 9/65), small ruminants (9.2%, *n* = 6/65), and other species (13.9%, *n* = 9/65).

#### 3.2.1. Veterinary Practitioners’ a Priori Knowledge on VHHM Projects and Training

The majority of respondents were unaware of VHHM projects (59.6%, *n* = 59/99). Of those participants (40%, *n* = 40/99) who reported knowledge of ongoing or recent projects related to VHHM, half (55.0%, *n* = 22/40) knew at least one out of the pre-selected and listed VHHM projects in the questionnaire. This was followed by participants (32.5%, *n* = 13/40) who did not know any of the listed VHHM projects and those who knew other projects that weren’t listed (12.5%, *n* = 5/40) (Table 2).

The majority of respondents were unaware of VHHM training programs (69.4%, *n* = 43/62). Out of those participants (31%, *n* = 19/62) who reported knowing ongoing or recent HHM training, a total of 22 training programs related to VHHM were identified, of which 18% (*n* = 4/22) led to an accredited specialist recognition. Half of the identified training programs (*n* = 11/22) were also accessible to other livestock professionals and 40% (*n* = 9/22) were also accessible to students. The majority (90%, *n* = 20/22) offered certification upon successful completion. Topics included disease prevention, nutrition, welfare, reproduction, and general herd management techniques for the benefit of both veterinarians and livestock producers (Table 3).

#### 3.2.2. Veterinary Practitioners’ VHHM Training Gaps and Needs

A total of *n* = 53 veterinarians assessed the training gaps on a four-point scale from ‘no gap’ to ‘very big gap’. The four categories are as follows: i. certificates and labeling, ii. soft skills, iii. showing benefits of VHHM to stakeholders, and iv. theory and practice. The biggest gap was indicated for i. labeling and certificates by 14.2% (*n* = 7/49) of the respondents and species specifically (*n* = 3 replied ‘I don’t know’); all veterinary practitioners reported at least 50.0% of a very big to a somewhat big gap in certification in VHHM training. Half of poultry veterinarians reported a small training gap (50.0%, *n* = 5/10) for certifications in VHHM training.

The next most prominent overall very big gap was indicated for ii. soft skills with 11.5% (*n* = 6/52) for all respondents (*n* = 1 replied ‘I don’t know’). The majority of pig veterinarians (62.5%, *n* = 5/8) reported a somewhat big gap in soft skills within VHHM training. Most poultry veterinarians (55.6%, *n* = 5/9) answered that there is a small gap in soft skills in VHHM training.

**Figure 2:**
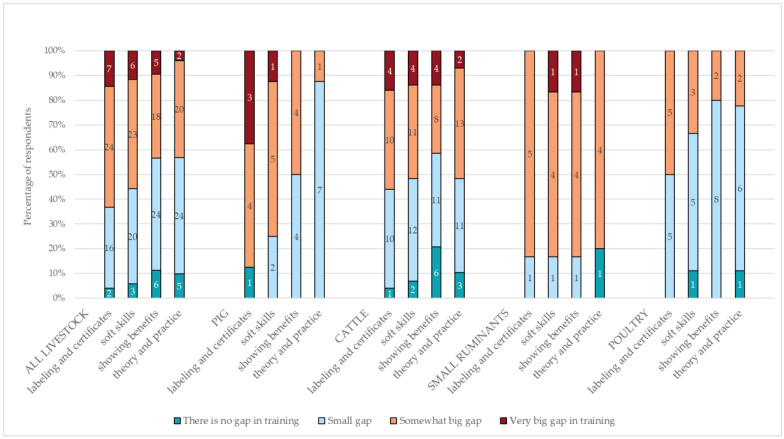
Reported perceived training gaps in VHHM training indicated species specifically by veterinary practitioners. The numbers within the bars indicate the number of respondents. Excluded answers “I do not know” are not shown.

For category iii. showing the benefits of VHHM to animal caretakers, an overall very big gap was indicated by 9.4% of the respondents (*n* = 5/53). The majority of poultry veterinarians (80.0%, *n* = 8/10) and half of pig veterinarians (50.0%, *n* = 4/8) reported the gap to be small. The least prominent overall very big gap was perceived for category iv. combining theory and practice solely by cattle veterinarians (3%, *n* = 2/51). A “mixed approach” in veterinary herd health management training is based on the acquaintance of theoretical knowledge and general principles with practical, case-specific ways to tailor the training experience of animal health practices to individual farm needs [14]. Overall data and species-specific data are in shown in Figure 2.

## 4. Discussion

The European veterinary profession has undergone significant transformation over the past few decades, including a demographic shift toward specialization in companion animal care [15]. This demographic change together with agricultural and socio-economic changes has had a substantial impact on veterinary education, training, and employment [4]. With fewer veterinarians attracted to livestock and fewer farms with larger herds, there is an increased emphasis on VHHM, including biosecurity, and preventive veterinary care. This shift necessitates a re-evaluation of veterinary curricula and training programs and a rethinking of how to perform routine animal health care in industrial settings to address emerging challenges [16,17]. Moreover, this evolving landscape has led to policy changes and the development of new frameworks, most importantly the AHL [12].

This study explored the current state of development of VHHM education and training by investigating veterinary undergraduate and postgraduate options, crosslinking veterinary policies and regulations.

### 4.1. The Importance of Teaching VHHM Is Increasingly Reflected in Undergraduate Veterinary Education

Veterinary education in Europe is highly harmonized through the ESEVT accreditation system run by the EAEVE and FVE [13,18], which builds upon the EU Directive 2005/36/EC [19]. Our results showed a high rate of VEE compliance with the ESEVT Standards requirements of VHHM education and that veterinary students have sufficient opportunities to learn about the different aspects of VHHM. The main difference between VEEs was the method of implementation and the depth of VHHM education. Most VEEs integrate the basic principles of VHHM into various courses within the core curriculum, such as animal husbandry, nutrition, epidemiology, and preventive veterinary medicine, while 27% in addition offer a dedicated VHHM course. While there is growing interest in companion animals and aquaculture, only 12% of VEEs currently provide shelter management or aquaculture health management training, representing a potential area for advancement. The current Commission Delegated Directive 2005/36/EC sets out minimum training requirements for veterinary surgeons in Annex V, Point 5.4.1. At the time of writing, in 2024, the EU aimed to revise the Directive to include VHHM in veterinary education requirements [20]. While Article 38(3)(c) refers to the prevention of animal diseases “whether considered individual or in groups”, the Annex of the Commission Delegated Directive does not reference VHHM specifically. Consequently, the EAEVE, the European Board of Veterinary Specialization (EBVS), the International Veterinary Students’ Association (IVSA), and the FVE, as members of the European Coordinating Committee on Veterinary Training (ECCVT), reviewed the draft Commission Delegated Directive and proposed changes in line with the ECCVT List of subjects and Day One Competences to include add ‘Animal health economics and practice management’ [18].

### 4.2. Post-Graduate VHHM Training Opportunities Leading to Certification Exist but Are Not Always Well-Known

While our study identified many VHHM training opportunities—many of which led to certification—the biggest gap was perceived for certification after completion of training activities by the participants of the survey. An exception to this was the group of poultry veterinarians, who reported smaller gaps in VHHM training certification, potentially due to integrated, company-provided training programs that support skill development more comprehensively. This suggests the need for awareness raising of more certified postgraduate VHHM training opportunities, including accessible CPD courses and structured further education for mixed practitioners leading to a diploma and/or recognizable expert status for clients and colleagues. National and board specialization offer in-depth training, and 20% of veterinarians in Europe reported to have followed national specialization and 7% reported to have followed an EBVS specialization [4]. However, the inherent commitment necessary to pursue specialization can be demanding for veterinary practitioners. The ECCVT has stated before that there is a need for middle-tier training, which is beyond the level of the first degree but below that of the specialist [21]. The presence of this middle tier of continuing education and qualifications also provides a structure within which veterinarians can complete their obligation to engage in lifelong learning to keep their knowledge and skills up to date. Already, a number of these middle-tier qualifications exist at a national level, based on the separate evolutionary pathways for the profession in individual member states before European systems were developed and agreed. Improved promotion of modular pathways may be an opportunity with mutual benefit for participants and providers of post-graduate VHHM training. In addition, the FVE recommends standardization of post-graduate structured CPD to facilitate recognition of skills and competences acquired by veterinarians throughout Europe [22].

### 4.3. Soft Skills and Communication Are Essential to Advise Animal Caretakers on the Benefits of VHHM

All veterinarians in our survey reported a notable gap in soft skills for VHHM training. It was shown previously that only 19% of recent veterinary graduates felt adequately prepared with the communication skills necessary to interact effectively with clients and colleagues [4]. As advisory roles become increasingly important within veterinary work, good communication is essential for ensuring adherence to recommendations and overall success in herd health management. Training programs in motivational interviewing, for example, have been shown to improve veterinarians’ communication skills [23]. Therefore, an important area for potential VHHM training improvement seems to be the development of soft skills among both veterinary students and practicing veterinarians.

For farmers to adopt a comprehensive VHHM plan alongside their veterinarians, it is crucial for them to perceive the tangible advantages, especially the economic benefits. As recognized by many veterinarians in our survey and shown before [24], there is an urgent need for additional research to show the cost-effectiveness of VHHM and preventive approaches to animal health problems. While numerous studies have quantified the economic benefits of preventive medicine in cattle [25,26,27], particularly for conditions such as mastitis and lameness, there is a lack of research focusing on other species, especially small ruminants. This scarcity is likely attributed to several reasons amongst which is the ‘prevention paradox,’ which complicates the quantification of economic benefits from preventive measures. These benefits are often difficult to quantify because they manifest as the absence of negative outcomes. Additionally, beyond these economic benefits, preventive veterinary services not only enhance animal health and welfare but also positively impact trade, pandemic prevention, and veterinary public health [28]. Consequently, there is a need for comprehensive research to explore the direct and indirect benefits of preventive veterinary medicine. While some benefits may not directly occur to individual farmers, they contribute significantly to societal welfare. This underscores the importance of societal support for preventive medicine, recognizing it as a public good that extends beyond individual farm economics.

### 4.4. Future Challenges and Opportunities: Looking Ahead to the Role of Technology in VHHM

VHHM relies on the thorough analysis of relevant data, ranging from diagnostics and productivity metrics to slaughterhouse data and real-time monitoring [29]. In recent years, technology, including artificial intelligence, has transformed and facilitated VHHM by enabling more evidence-based treatments and predictive capacities. While studies have shown that veterinarians are keen to leverage these data to enhance their VHHM practices, challenges with respect to data management remain, especially for independent veterinarians looking after different livestock species in non-integrated systems [30]. While this was not reflected in the survey questions, it will be essential to further work on better VHHM data management, availability, and access options and train veterinarians on how to operate and educate them on the proper use of these advancements.

## 5. Conclusions

In summary, due to socio-economic, professional, legislative, and ethical structural changes, VHHM will continue to gain increasing importance in the future. While there are strong educational foundations for VHHM at the undergraduate veterinary education level, there is a clear need to expand and further promote certified postgraduate training, especially for established veterinary practitioners operating in multi-species settings. Additionally, greater emphasis on soft skills training was identified as important for both under- and postgraduate training to ensure the future success of veterinarians in this field. The findings also underscore the urgent need for robust economic evaluations of preventive strategies across livestock species to allow veterinarians to better demonstrate VHHM’s direct and indirect benefits to farmers. Veterinarians must also receive training on how to operate, use, and instruct other allied professionals on how to properly utilize new technological advancements.

## Figures and Tables

**Table 1:** Data extracted on veterinary herd health management by type of education and per species from the evaluation reports of 41 VEEs visited between 2018 and 2022.

Veterinary Herd Health Management by Type of Education	Species Category	Number of VEE Covering the Type and Species/All Visitations % VEE
Theoretical training	Terrestrial livestock	*n* = 37/41 90%
Practical training and rotations		*n* = 40/41 98%
External practical training		*n* = 33/41 81%
Mobile/traveling veterinary practice		*n* = 36/41 88%
Voluntary options for herd health management training		*n* = 17/41 42%
Logging/reports of formal herd health management training		*n* = 37/41 90%
Equine herd health management	Equines	*n* = 17/41 42%
Shelter/kennel management	Companion animals	*n* = 5/41 12%
Aquaculture health management	Aquatic livestock	*n* = 5/41 12%

**Table 2:** Selected examples of EU-funded projects on topics related to Herd Health Management. Projects for which websites were not available at the time of writing were excluded. (V—veterinarians, L—livestock professionals, O—others, and NA—not available/specified). “Listed projects” are those pre-selected and provided in question, while “mentioned projects” are those brought up by the respondents in their answers.

Project	Name of Project	Species	For Whom?	Is It Active? (Running at the Time of the Survey)	Do They Offer Educational Activities?
LISTED	Armenta APT	Cattle	N	No	No
	Cure4Aqua	Aquatic livestock	V, L, O	Yes	Yes
	EIP-AGRI	Terrestrial livestock	L	No	Yes
	LINKTADS	Terrestrial livestock	V, O	No	Yes
	AGRICAM	Cattle	V, L	Yes	Yes
MENTIONED	DECIDE	Terrestrial and aquatic livestock	V, L, O	Yes	Yes
	Code:Re-farm	Poultry and goats	N	No	No
	BROILERNET	Poultry	V, L, O	Yes	Yes
	SOLID	Cattle	V, L, O	No	Yes
	COST Action BETTER	Terrestrial livestock	V, O	Yes	Yes

**Table 3:** Identified organizations which providedtraining on topics related to Herd Health Management mentioned by survey participants (V—veterinarians, L—livestock professionals, and S—students).

Training Provider and Link	Species	Format of Training	Target Audience	Educational Level	Certificate Provided Upon Successful Completion	Training Currently Available?
WAVMA CertAqV	Aquatic livestock	Online and face to face	V, S	Middle-tier	Yes	Yes
PerFormFish		Online	V, L, S	Middle-tier	Yes	Yes
European College of Aquatic Animal Health		Online and face to face	V	Accredited Specialist	Yes	Yes
Animal Health Ireland	Terrestrial livestock	Online and face to face	V, L	Middle-tier	Yes	Yes
DISARM		Online	V, L	Middle-tier	No	Yes
FarmSkills4Vets		Online	V, S	Middle-tier	Yes	Yes
BioCheck		Online	V, L, S	Middle-tier	Yes	Yes
University of Illinois Urbana-Champaign		Online	V, L, S	Middle-tier	Yes	Yes
Aberystwyth University		Online	V, L, S	Middle-tier	Yes	Yes
KFL		Online	V	Middle-tier	Yes	Yes
FAO elearning Academy		Online	V, L	Middle-tier	Yes	Yes
EuFMD	Cattle	Online	V, L, S	Middle-tier	Yes	Yes
Pennsylvania State University		Online	V, L, S	Middle-tier	Yes	Yes
European College of Bovine Health Management		Online and face to face	V	Accredited Specialist	Yes	Yes
Flock Health Clubs	Small Ruminants	Online and face to face	V, L	Middle-tier	No	Yes
European College of Small Ruminant Health Management		Online and face to face	V	Accredited Specialist	Yes	Yes
Faculty of Veterinary Medicine, University of Zagreb	Pigs	Online and face to face	V, S	Middle-tier	Yes	Yes
European College of Porcine Health Management		Online and Face to face	V	Specialist	Yes	Yes
FJØRFESKOLEN	Poultry	Face to face	L	Middle-tier	Yes	No
LXII CONVEGNO ANNUALE SIPA		Face to face	V	Middle-tier	Yes	No *
WVPAC 2023		Face to face	V	Middle-tier	Yes	No
European College of Poultry Veterinary Science		Online and face to face	V	Accredited Specialist	Yes	Yes

* post-activity after symposiums.

## Data Availability

The original contributions presented in this study are included in the article/Supplementary Materials; further inquiries can be directed to the corresponding author.

## Supplementary Materials

The following supporting information can be downloaded at: https://www.mdpi.com/article/10.3390/vetsci11110528/s1. Table S1: CA20103 BETTER Task 4.3 online survey questionnaire.
